# Exploring structural heterogeneity and organizational patterns of tourist flow networks among motivational subgroups

**DOI:** 10.1371/journal.pone.0323558

**Published:** 2025-06-02

**Authors:** Xumei Pan, Ting Liu, Lixia Yan

**Affiliations:** School of Geographical Science, Shanxi Normal University, Taiyuan, Shanxi, China; Politecnico de Milano, SPAIN

## Abstract

Tourists’ motivation is a key factor influencing their spatiotemporal behavior patterns. However, studies analyzing the spatial movement patterns of tourists with different motives are scant. In this study, the travel diaries of 4,306 tourists were employed as the primary data source, and the Louvain algorithm was applied to identify tourist flow networks with different motives; moreover, the complex network method was utilized to analyze the hierarchical structure and heterogeneity of the organizational patterns of tourist flow networks with different motives. The analysis revealed the following main conclusions. (1) There are three types of tourism flow networks with different motivations: exploring nature, exploring culture and nature-culture motivations. (2) All types of motivated tourist flow networks exhibit significant node hierarchies. The core nodes in natural network are the most controllable, whereas those in natural-cultural network are less controllable and more evenly connected. (3) Node connectivity strength decays significantly with distance. Geographic constraints exert the greatest impact on natural network. (4) Different-motivated tourist flow networks present a Multi-scale Hierarchical Nested Pattern. Tourists gather in multi-core cultural networks and multi-core natural-cultural networks, using them as a base to spread to neighboring regions. They shift between core nodes and use new cores as a base for tours. This study enriches the theoretical system of tourist behavior research and provides effective practical recommendations for the Shanxi tourism industry to improve tourists’ travel experience.

## 1 Introduction

Among the factors influencing tourists’ spatiotemporal behavioral patterns, motivation is considered a key factor—directly associated with the type of destination that tourists select for their trips and the mobility between destinations [1]. Specifically, motivation refers to the intrinsic drive that encourages tourists to engage in tourism activities, such as relaxation, recreation, cultural experiences, and socialization [2]. Tourists’ motivations directly influence the types of destinations that they select; for example, tourists seeking relaxation may select destinations with natural beauty, whereas tourists interested in culture may select destinations rich in history and culture [3–5]. Additionally, motivation influences tourists’ movement between destinations. For example, tourists motivated to experience history and culture are highly likely to visit multiple destinations of the same type in a single trip [6].

Therefore, parsing the spatial movement patterns of tourists with different motivations is of practical significance [7,8]. First, it can help understand tourists’ mobility probability between destinations of the same type, which can be utilized to design theme-based tourism routes, tourism marketing, and destination recommendations [9,10]. Second, it can help classify multiple destinations in a region into associations based on tourists’ needs [11,12]. Regional governments can utilize the node hierarchy in associations to develop tourist diversion strategies to alleviate environmental and social issues caused by overtourism and congestion [13]. Furthermore, integrated marketing strategies can be developed based on the type of association to increase the cumulative attractiveness of tourist flow clusters [14].

However, research analyzing the spatial movement patterns of tourists with different motivations is limited. Current studies predominantly focus on exploring the influence of factors such as distance (physical, cultural, and transportation distance), length of stay, and tourists’ attributes (form of tourism organization, place of residence, age, etc.), on the spatial movement patterns of tourism [12,15–21]. These studies are primarily based on tourism big data (e.g., Ctrip.com) [22]. These big data platforms ask users to upload personal attribute information regarding the travel process, thus providing immediate and effective information for analyzing the differences in the behavioral patterns of individuals with different attributes [21]. However, no platform requires tourists to upload their motivations for traveling; consequently, research on the differences in the spatial movement patterns of tourist subgroups based on motivations is scant, thereby limiting our understanding of the spatiotemporal characteristics of different social groups [23,24].

Understanding the spatial movement patterns of subgroups of tourists with different motivations is of theoretical and practical significance. In order to achieve this goal, there are two key issues: first, how to obtain a tourist flow network that reflects the motivations of tourists; second, how to parse the hierarchical structure and patterns of tourist flow networks among motivational subgroups. In response to the first question, the relevant literature revealed that tourists’ motivation to travel can be inferred inversely by dissecting the types of destinations that they visit. As Xue [19] highlighted, the types of destinations visited by tourists reflect their traveling motivations. Based on the types of destinations selected by tourists and their mobility trajectories between destinations of the same type, the spatial mobility patterns of tourist groups with that motivation can be accurately identified [25]. That is, when all destinations in an identified tourist flow association belong to the same type, the association can be further analyzed to derive the spatial mobility patterns of that motivated tourist subgroup. The Louvain algorithm is used to parse the tourist flow networks and found that the identified clusters exhibited internal consistency. The Louvain algorithm—a community discovery algorithm based on modularity optimization—has become a powerful tool for addressing community discovery problems in large social networks owing to its high efficiency, scalability, ability to accurately identify the community’s structure, and widespread practical applications [26]. In response to the second question, the complex network algorithm is chosen in this study to parse the hierarchical structure and patterns of tourist flow networks. The complex network analysis method involves modeling a complex system as a network, and an in-depth analysis of its topology can reveal hidden organizational patterns, hierarchical relationships, and key nodes in the complex system. Thus, it has become a typical paradigm for flow space research [27].

In this study, we build a tourist flow network based on the online travel diaries of Ctrip, Mafengwo, Qunar, and Lvmama websites; apply the Louvain algorithm to parse Shanxi tourists’ spatial behavioral network; further, the social network algorithm was applied to parse the hierarchical structure of the tourist flow network with different motives and organizational pattern of the heterogeneity. This study’s main contributions are as follows: (1) Unlike existing studies, we employed motivation as a segmentation dimension to explore the differences in the spatiotemporal behavioral patterns of different tourist subgroups, thus providing novel insights into tourist behavior research. (2) We quantified the differences in flow patterns across different motivational clusters. The hierarchical structure, geographic constraints, and aggregation-diffusion effects of the mobility networks of tourists with different motivations were compared using various indicators, such as node centrality, distance decay curves, and structural holes. Our findings offer effective practical recommendations for the Shanxi tourism industry to improve tourists’ travel experiences.

The remainder of this paper is organized as follows: Section 2 reviews relevant research and highlights this study’s necessity and innovativeness in complementing extant theories of tourist behavior research. Section 3 elucidates the importance of the model employed in this study, the specific research questions that the model addresses, and how the study data were collected and processed. Section 4 discusses this study’s results in depth. Sections 5 and 6 summarize this study’s importance and limitations and present future research directions.

## 2 Literature review

Numerous studies have discussed the spatiotemporal patterns of tourists across diverse segments, particularly demographic characteristics [15,17,18,20]. Xu et al. [25] compared the differences in the spatiotemporal behaviors of resident and non-resident tourists and found that locals visited nature parks and hutongs on weekends, whereas foreign tourists visited popular attractions in the city center during holidays. Tian et al. [7] observed differences in mobility indicators, temporal patterns, and spatial patterns between subgroups owing to differences in sex, age, and place of origin. Older tourists exhibit the highest enthusiasm for traveling, whereas middle-aged tourists exhibit the lowest enthusiasm. Female tourists exhibit earlier departure times and longer travel times than male tourists. The spatial structure of neighboring tourists is centered on train stations, whereas that of non-neighboring tourists is centered on airports. Additionally, the behavioral patterns of first-time and returning tourists differ considerably, as indicated by first-time tourists’ likelihood of making long trips away from the hotel that they are staying at, whereas returning tourists make multiple short trips intermittently to and from the hotel and tourist attractions [1]. Additionally, the place of origin affects tourists’ behavioral patterns. Liu et al. [17] reported that among tourists visiting Hong Kong, tourists from Japan preferred “heritage centers” and “art galleries/performance venues,” whereas tourists from Thailand preferred “historical sites” and “street markets.”

Moreover, travel distance is a key factor influencing tourists’ behavioral patterns [19]. Tourists’ understanding of distance extends beyond physical distance to cover relative distance cost, time, and cultural differences. These dimensions precipitate differences in tourists’ spatiotemporal behavioral patterns [16]. Liu et al. [28] utilized Dunhuang, an important tourist destination in Northwest China, as a case study to explore the tourist flow network under different transportation modes and found that tourists’ travel behavior patterns exhibit differences under different transportation mode networks. Additionally, economic distance influences tourists’ travel patterns, as tourists may be “more willing to visit and stay longer in destinations with similar infrastructural and service endowments because it reduces their perceptions of the risks involved in traveling to such destinations.” Jackman et al. [29] observed that leisure tourists’ length of stay in Barbados decreases as economic distance increases.

Motivation has been considered a key factor in tourist mobility patterns. Leisure travelers are more likely to explore destinations than business travelers, and individuals tend to spend less time visiting friends and relatives and more with family [1]. When traveling, they are likely to visit areas that are not primarily identified as tourist nodes. Masiero and Zoltan [6] examined motivation’s effect on the number of scenic spots and found that tourists who traveled to historical places and tried new things were more likely to engage in extensive destination travel, whereas those interested in experiencing scenery, nature, or physical activity exhibited less extensive but more intense spatial movement. Special-interest tourists limit their movements to activities related to a specific visit, whereas generalist sightseeing tourists travel more extensively, with no discernible pattern. Additionally, much current research emphasizes motivation’s influence on tourists’ behavior. Cezar Morar [21] examined changes in tourists’ travel behaviors and preferences after COVID-19 and found that the primary travel motivation was pleasure-seeking and that the fear of travel and perceived infection risk at the destination were important factors limiting their travel. Extant studies have less compared behavioral differences between motivated travel subgroups, which may have resulted in a limited understanding of the differences in travel mobility patterns [24].

In sum, tourism motivation is a key factor in segmenting tourists’ behavioral patterns. However, compared to the research on tourism distance and tourist attributes, relevant studies are still exploring travel motivation’s influence on tourism behavior. Summary and comparative studies examining the corresponding spatial behavior patterns remain scarce, and additional investigation is needed to understand their heterogeneity.

## 3 Methodology

### 3.1 Study area

**Fig 1:**
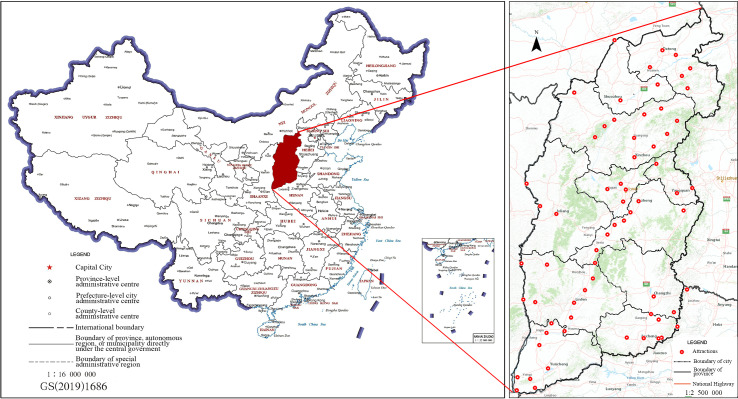
Location and tourist attractions of Shanxi province. This map’s political boundaries are drawn based on the standard maps from China’s Ministry of Natural Resources and Department of Natural Resources of Shanxi Province (Surveying and mapping approval No. GS (2019)1686 and No. JS (2021)005). The base map remains unaltered. The same applies to the digital elevation model derived from the U.S. Geological Survey (http://eros.usgs.gov/).

Located in the Yellow River Basin and Eastern Loess Plateau of China (**Fig 1**), Shanxi Province has a total area of 156,600 square kilometers. Shanxi Province is a parallelogram inclined to the southwest of the northeast. The complex topographical conditions and evident transitional climate of Shanxi Province nurture colorful and characteristic natural attractions, including famous mountains and rivers, karst caves and rocks, clear springs and lakes, rapids and waterfalls, and precious creatures. Additionally, Shanxi Province—among the birthplaces of Chinese civilization—was China’s political, economic, and cultural center. Numerous high-quality historical and cultural tourism attractions exist, which hold a prominent position in China. In 2019, Shanxi Province attracted 730 million visitors, and visitors’ expenditures totaled 802.69 billion yuan. Therefore, Shanxi’s tourism industry plays a pivotal role in the local economy and was, thus, selected as a representative case to analyze the spatial patterns of tourist flow. The applied method can, naturally, be expanded to analyze other provinces with similar data availability.

### 3.2 Data collection

Previous studies have indicated that online travel diaries document tourists’ spatiotemporal trajectories in real time and can be used to study the spatial patterns of tourists’ mobility [23,27,30]. Considering the differences between user groups, we utilized the online travel diaries obtained from Ctrip, Mafengwo, Qunar, and Lvmama websites as the data These four websites—all of which have large user groups and extremely high user activity—fully reflect the travel behaviors and preferences of online travel users, which helps analyze and examine related issues in depth [31,32]. We collected 4,306 online travel diaries published from 1999 to 2021; our data collection period extended from April 7 to May 3, 2024. We refer to Bindan Zeng’s [28] data preprocessing process—that is, eliminating duplicates and advertisements, organizing destination visit routes, and harmonizing destination names—to ensure its application to subsequent studies.

Destination types reflect tourists’ motivation to visit [29]. If multiple destinations of the same type attract the same tourists, parsing the probability, frequency, and significant nodes of tourist flows between such destinations reveal these motivated tourists’ behavioral patterns [27]. Therefore, it is reasonable to speculate that when all destinations in an identified tourist flow association are of the same type, the association can be further analyzed to derive the spatial movement patterns of that motivated tourist subgroup. Thus, the identification of destination types is critical. This study adopted the National Tourism Resources Survey, Classification, and Evaluation Standard (GB/T 18972–2017)—currently the most authoritative classification standard for tourism destinations in China. Based on this standard, the 137 tourist destinations identified were categorized and their types were determined, of which 101 were cultural and 36 were natural scenery.

### 3.3 Methods

#### 3.3.1 Community detection algorithm.

Tourists’ motivations can be inferred inversely based on the type of destination that they visit [19]. Based on the types of destinations selected by tourists and their mobility trajectories between destinations of the same type, the spatial mobility patterns of tourist groups with that motivation can be accurately identified [33]. That is, when all destinations in an identified tourist flow association belong to the same type, the association can be further analyzed to derive the spatial mobility patterns of that motivated tourist subgroup.

In this study, we utilized the Louvain algorithm to parse the tourist flow networks and found that the identified clusters exhibited internal consistency. The Louvain algorithm—a community discovery algorithm based on modularity optimization—has become a powerful tool for addressing community discovery problems in large social networks owing to its high efficiency, scalability, ability to accurately identify the community’s structure, and widespread practical applications [34]. Specifically, (i) the Louvain algorithm has a time complexity of O(nlogn), which is more efficient in managing large-scale networks than other algorithms, such as the Girvan-Newman algorithm O($n^{3}$); moreover, it can quickly complete the community discovery task. (ii) The Louvain algorithm can quickly handle all types of large-scale weighted networks, including directed and undirected networks, and adapt to the needs of network structure analysis in diverse scenarios [32]. (iii) The Louvain algorithm effectively optimizes the modularity and accurately identifies the community structure in the network through a two-phase iterative process, that is, node moving and community merging. Even noisy and data-deficient networks can reduce the local perturbations and stably discover the main community structures using a greedy strategy. This process enables the Louvain algorithm to accurately recognize community structures [34]. The Louvain algorithm is widely used in numerous fields, including sociology, communication, and biology, owing to its community discovery ability [13].

In this study, referring to Emmons et al.’s method [35], we set the number of repetitive iterations to 500 times and performed multiple Louvain algorithm runs to obtain the maximum degree of modularity, which is the most stable result of community delineation. Step 1: Construct a 137 × 137 weight matrix, wherein the attractions correspond to each node of the weight matrix and each node is considered a separate community. Thus, the initial number of communities equals the number of nodes. Step 2: Iterate over each node d ^_α in the weight matrix and compute the modularity gain ΔQ of the node from the current community to all neighboring communities. If maxΔQ >0, the node is included in the community wherein the neighboring node with the maximum relative modularity is located; otherwise, it remains unchanged. Step 3: Step 2 is repeated until the communities of the network no longer change. Step 4: The communities generated in Steps 2 and 3 are compressed, and each community is updated as a new supernode by setting the sum of the total weights of the connected edges between the communities as the self-loop weights between the new nodes. At this point, a new weight matrix is generated, and the new overall modularity of the network is computed Q. Step 5: Steps 1–4 are repeated until the modularity of the entire network no longer changes. Again, in this study’s context, attractions can eventually find the tourist clusters to which they belong according to the tourists’ motivations. The calculated final modularity Q is 0.797829 > 0.5, which is a more appropriate community segmentation result [34], with tight connections within clusters and sparse connections between clusters.


$$\Delta Q=\left[\frac{\sum in+2{\hat{d}}_{\alpha ,in}}{2m}-\left(\frac{\sum tot+{\hat{d}}_{\alpha }}{2m}\right)\right]-\left[\frac{\sum in}{2m}-{\left(\frac{\sum tot}{2m}\right)}^{2}-{\left(\frac{{\hat{d}}_{\alpha }}{2m}\right)}^{2}\right]$$ (1)

where ∑in is the total degree of all edges in community c, ∑tot is the total degree of all edges to nodes in community c, and ${\hat{d}}_{\alpha ,in}$ is the number of edges of all nodes in community c, and m is the total number of edges in the network.

#### 3.3.2 Complex network algorithm.

The complex network analysis method involves modeling a complex system as a network, and an in-depth analysis of its topology can reveal hidden organizational patterns, hierarchical relationships, and key nodes in the complex system. Thus, it has become a typical paradigm for flow space research [36]. Compared to traditional analysis methods, it can adapt to dynamically changing complex network systems; accurately identify important nodes, hierarchical structures, and associated paths playing a decisive role in the system’s overall function and stability; and is suitable for dealing with complex relational networks that are prevalent in the real world, such as transportation, biological, and social networks [37]. This method fits this study’s objective, which is to deeply explore the hierarchical structure between nodes within the community and reveal the intrinsic organizational laws and spatial patterns of tourism flow networks with different motivations.

Therefore, we applied this method to examine the communities identified using the Louvain algorithm in depth and analyzed the hierarchical structure of nodes and communities from the three dimensions of “point, line, and surface”—as presented in Table 1. At the “point” level, we focused on node centrality, including degree, proximity, and intermediate centrality, which measure the importance and influence of nodes in the network from different angles. Degree centrality reflects the number of direct connections of a node; proximity centrality reflects a node’s proximity to other nodes; and intermediate centrality reveals a node’s key role as a bridge in the network. At the “line” level, network centrality and association strength are utilized to measure the degree of connectivity and information flow efficiency of the network as a whole. At the “surface” level, structural holes theory is applied to reveal the connectivity failures and structural holes between connections in the network through the indicators of effective size and constraints, reflecting the network’s stability and vulnerability. This multidimensional analysis framework enabled us to comprehensively and systematically analyze the hierarchical structure of nodes and communities.

**Table 1:** Metrics of the complex network algorithm.

	Indicator	Formula	Explanation
Nodes centrality	Degree centrality	$\begin{array}{c} C_{AD}(i)=\sum_{j}^{n}\;r_{ij} \end{array}$	$r_{ij}$ represents the frequency of tourists visiting nodes i to j simultaneously.
	Closeness centrality	$C_{C}(i)=\frac{1}{\sum_{j=1}^{\prime }\;d(i,j)}$	d(i,j) represents the shortest distance that a node can travel to each of the other points in its connectivity component.
	Between centrality	$\begin{array}{c} C_{B}\left(n_{i}\right)=\sum_{j}^{\prime }\;\sum_{k}^{\prime }\;\frac{g_{jk}(i)}{g_{jk}} \end{array}$	$g_{jk}$ represents the number of paths that the traveler takes from nodes j to k; $g_{jk}(i)$ is the number of paths that pass through point i.
Clusters centrality	Degree centrality	$C_{D}=\frac{\left[\sum_{i=1}^{n}\;\left(C_{ADmax}-C_{AD}(i)\right)\right]}{n^{2}-3n+2}$	$C_{D}$ represents the degree centrality of the nodes in the cluster, and n represents the number of nodes.
	Closeness centrality	$\begin{array}{cc} & C_{C}=\frac{\sum_{i=1}^{g}\;\left[C_{C}\left(n^{*}\right)-C_{C}\left(n_{j}\right)\right]}{[(n-2)(n-1)]/(2n-3)} \end{array}$	$C_{C}\left(n^{*}\right)$ represents the maximum value of near centrality of nodes in this cluster.
	Between centrality	$\begin{array}{cc} & C_{B}=\frac{2\sum_{i=1}^{g}\;\left[C_{B}\left(n^{*}\right)-C_{B}\left(n_{i}\right)\right]}{\left[(n-1{)}^{2}(n-2)\right]} \end{array}$	$C_{B}\left(n^{*}\right)$ is the maximum value of the degree centrality of all nodes in the cluster.
Structural holes	Effective size	$\begin{array}{c} ES_{i}=\sum_{j}^{n}\;\left(1-\sum_{q}^{n}\;p_{iq}m_{jq}\right),q\neq i,j,\text{ where }\\ p_{iq}=\frac{\left(z_{iq}+z_{qi}\right)}{\sum_{j}^{n}\;\left(z_{ij}+z_{ji}\right)},i\neq j\\ m_{jq}=\frac{\left(z_{jq}+z_{qj}\right)}{\max\left(z_{jk}+z_{kj}\right)},j\neq k \end{array}$	$p_{iq}$ is the number of connections between nodes 𝑖 and *q* divided by the maximum number of connections between node 𝑖 and one of the other nodes; $m_{jq}$ is the strength of the relationship edge between network members *q* and 𝑗.
	Constraint	$\begin{array}{c} {CT}_{i}=\sum_{j}^{n}\;{\left(p_{ij}+\sum_{q}^{n}\;p_{iq}p_{qj}\right)}^{2},q\neq i,j \end{array}$	$p_{ij}$ is the number of connections between nodes i and j divided by the maximum number of connections between node 𝑖 and one of the other nodes.

## 4 Results

### 4.1 Attractions clustering and category distribution

#### 4.1.1 Attractions clustering.

**Fig 2:**
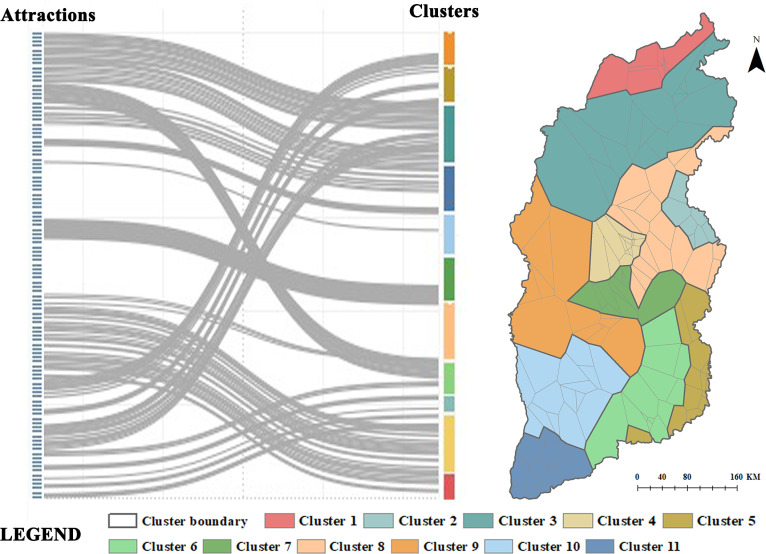
Attractions clustering according to frequencies of tourist flow directions. This map’s political boundaries are drawn based on the standard maps derived from the Department of Natural Resources of Shanxi Province (Surveying and mapping approval No. JS (2024)010).

Using Louvain’s community discovery algorithm, we obtained 11 “tourist stream communities”, as presented in **Fig 2**. From the delineation results, the clusters were characterized by the following features: (1) Geographic connectedness. Attractions within the clusters were geospatially neighboring or close to each other. Each cluster had a dominant spatial area formed by the agglomeration of geographically neighboring tourist attractions—predominantly because of the double constraints of natural conditions. (2) Discontinuity in some clusters. Some special communities were distributed across several counties and cities simultaneously, such as Cluster 5. This phenomenon indicates that the limiting effect of spatial constraints on tourists was significantly weakened. (3) Clustering across cities and counties Most clusters did not comprise a single county or city; instead, they were regional functional clusters combining multiple counties and cities, suggesting that the links between tourist attractions have been gradually breaking owing to the limitations of administrative divisions.

#### 4.1.2 Category distribution.

**Fig 3:**
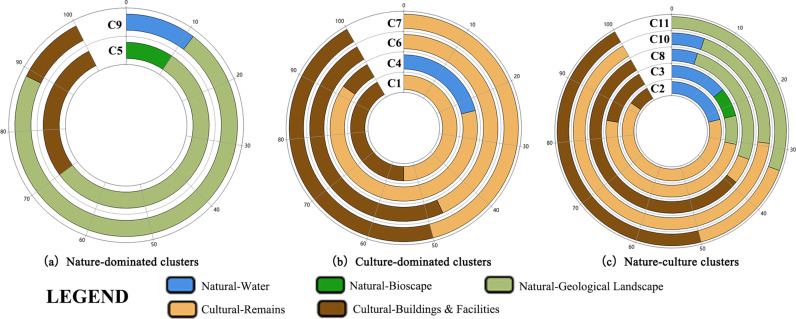
Cluster categorization based on attraction type percentage.

Based on the tourism attraction classification scheme in the standard of “Classification, Investigation, and Evaluation of tourism resources,” we divided the identified 11 clusters into three categories—namely, nature-dominated, culture-dominated, and nature-culture clusters—as presented in Fig 3. The nature-dominated clusters were Clusters 5 and 9. Over 70% of the attractions in these two clusters were natural landscapes. Notably, C1, C4, C6, and C7 were dominated by cultural attractions, at over 75%. Further, C2, C3, C8, C10, and C11 were dominated by natural and cultural attractions. The number of humanistic and natural attractions in these associations was relatively balanced. They were categorized into the nature-culture cluster.

### 4.2 Heterogeneity in cascading structure of tourism clusters

#### 4.2.1 Heterogeneity in hierarchical structure among cluster nodes.

**Fig 4:**
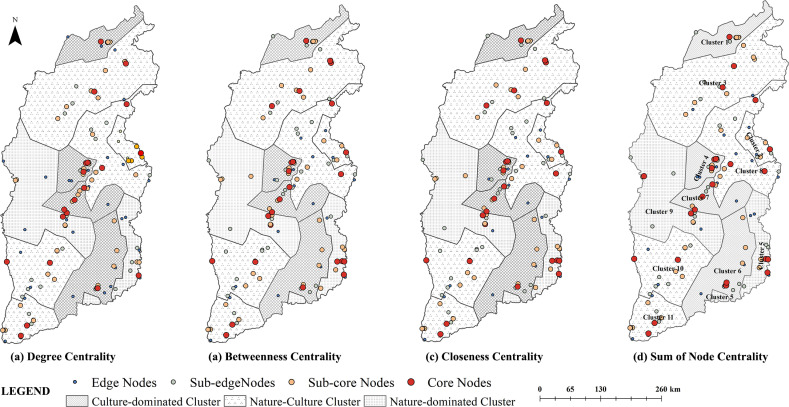
Hierarchies of nodes centrality among clusters.

By superimposing the values of degree, betweenness, and closeness centrality, we obtained the total centrality and importance status of each node. We stratified these nodes using the natural break method (**Fig 4**). Some nodes in the network of tourism flows in Shanxi developed into the core of the network of tourism flows and continuously strengthened their position through polarization. The other nodes were highly dependent on the core nodes.

The network had four types of nodes—specifically, core, sub-core, sub-edge, and edge nodes, which form a multilayered structure. **Fig 4** and **Table 2** present two types of core nodes as follows: (1) nodes located in the network’s center, with strong attraction and strong ability to collect and control tourists (e.g., Yungang Grottoes, Hanging Temple, Yingxian Wooden Pagoda, Wutai Mountain, and Pingyao Ancient City). (2) nodes in an important position in the transportation network, including an important transit and distribution center (e.g., the Jin Temple). Sub-core nodes predominantly could attract tourists and exhibited strong connections with other nodes (e.g., Luya Mountain, Ding Village, and Xiangyu Ancient Town). Sub-edge nodes were located within the radiation area of the core nodes, with fewer arriving tourists (e.g., Jinhua Palace, Datong Tulin, and Pool God Temple). Edge nodes were primarily geographically remote and limited by long distances and transportation costs (e.g., Dazhou Village and Wenfeng Pagoda). A certain degree of randomness exists in them joining the cluster.

**Table 2:** Nodes hierarchy and visit probability among clusters.

Category	Cluster	Core nodes		Sub-core nodes		Sub-edge nodes		Edge nodes	
		No.	Visit probability (%)	No.	Visit probability (%)	No.	Visit probability (%)	No.	Visit probability (%)
Nature-dominated	C 5	3	49.495	3	20.202	3	24.242	4	6.061
	C 9	2	41.164	2	29.032	6	27.572	1	1.935
Culture-dominated	C 1	1	19.841	4	43.651	5	27.778	4	46.154
	C 4	3	56.589	6	24.031	5	10.078	4	9.302
	C 6	2	30.667	3	26.000	5	19.333	3	24.000
	C 7	3	53.964	2	28.645	2	15.601	3	1.790
Nature-culture	C 2	1	25.714	2	42.857	3	14.286	3	17.143
	C 3	2	50.962	6	23.077	5	20.673	4	5.288
	C 8	3	32.787	3	26.230	4	18.033	6	22.951
	C 10	2	34.591	5	48.428	4	6.918	3	10.063
	C 11	2	13.33	4	35.398	7	34.513	2	7.080

Natural clusters exhibited highly controllable core nodes, whereas natural-cultural clusters had fewer controllable core nodes and maintained more balanced connections. By comparing the results of betweenness centrality and visit probabilities, we found that nature-dominated clusters had the highest concentration of tourist flows, as influenced by transportation accessibility. Cluster 5 (nature-dominated) had the largest standard deviation in betweenness centrality. The arrival probability of its core nodes was high, accounting for 49.495%. Cluster 9 (nature-dominated) had a relatively small standard deviation of betweenness centrality but comprised the most nodes with a mediated centrality of 0. The degree of agglomeration of the culture-dominated clusters was relatively low. The core nodes in these communities had a significantly larger agglomeration effect than spillover effects on tourists. Nature-culture clusters, which had fewer controlling core nodes and relatively balanced connections, exhibited a lower mean of mediated centrality variance, a lower number of 0 nodes, and a relatively low arriving probability of core nodes.

#### 4.2.2 Heterogeneity in geographic attenuation effect of links between cluster nodes.

**Fig 5:**
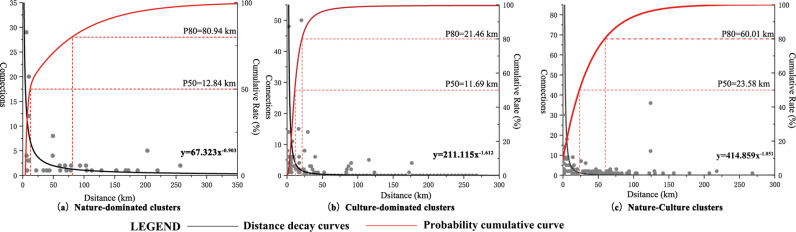
Distance decay curves and probability cumulative curves of three categorization clusters.

Connections between nodes are characterized by territoriality. Nodes close to each other were extremely closely connected. As the tour line’s length increased, the travel cost gradually increased, and the probability of generating a long-distance tour line pattern decreased accordingly (**Fig 5**).

Comparing the distance decay curves of tourist flow within the three cluster categories, we found that geographic constraints had the most significant impact on nature-dominated clusters. The frequency of the attraction association of nature-dominated clusters at the distance of 4–20 km was significantly higher, with an exponential distribution (y = 67.323x^-0.903^), and the distance attenuation index was -0.903. The frequency of the attraction association of culture-dominated clusters was significantly higher at the distance of 1–21 km. Its distance attenuation index reached -1.612. The frequency of the attraction association of nature-culture clusters was significantly higher at the 60 km distance, expressed as an exponential distribution (y = 414.859x^-1.851^). This suggests that in designing tourism routes, increasing the number of diverse destinations was conducive to prolonging tourists’ travel time.

By comparing the probability cumulative curves of the three cluster categorizations, we found that the three types of associations had large differences, with 80% of the cumulative curve as the boundary. The nature-dominated cluster’s tourism flows were concentrated in the 0–80 km section. Culture-dominated clusters were concentrated in the 0–21.46 km section. The nature-culture cluster was concentrated in the 0–60 km section. From the perspective of the technical and economic characteristics of the transportation modes, the road transportation’s economic radius was 0–200 km. The correlation of tourist flow decreased with the distance.

#### 4.2.3 Heterogeneity of agglomeration and diffusion effects among clusters.

**Fig 6:**
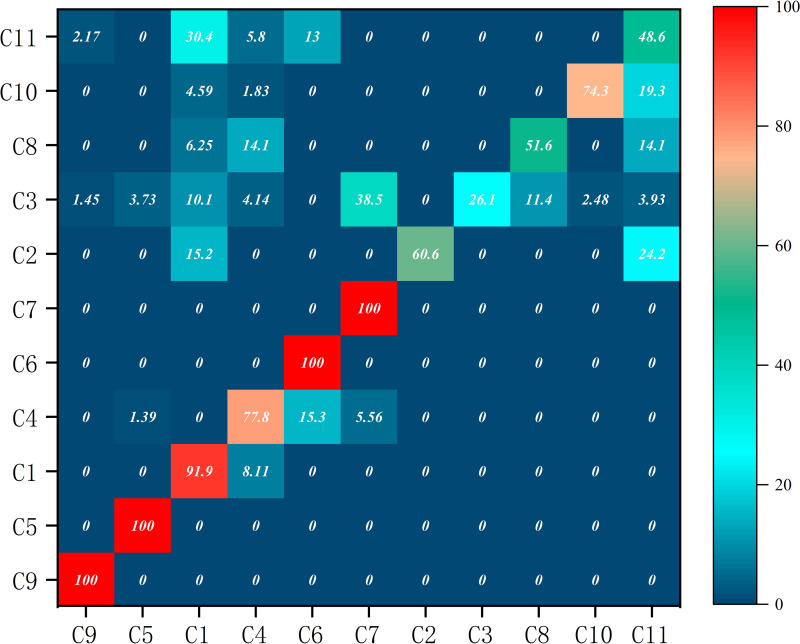
Transport transition probability between clusters.

Regarding the degree of association with other communities and probability of tourist transit, nature-culture clusters > culture-dominated clusters > nature-dominated clusters (**Fig 6**). Specifically, in nature-dominated clusters, links of Clusters 9 and 5 were highly concentrated within the community, with fewer tourists using them for transit or travel to other communities. The culture-dominated clusters had some exchanges with other types of clusters, especially Cluster 4. Nature-culture clusters had the highest likelihood of tourists transferring, with tourists from Cluster 3 and all other clusters except Clusters 6 and 2. This may be because nature-culture clusters were predominantly located in economically developed, industry-complementary, road-network, hierarchical, and well-connected areas.

Further analysis of the hierarchical structure of the clusters revealed (**Table 3**) that both composite and humanistic communities developed controlling nodes, which were in an important position in Shanxi’s tourism flow network, while nature-dominated clusters were relatively weak. Among nature-culture clusters, Clusters 11 and 3 had higher values for degree centrality, intermediary centrality, effectiveness, and efficiency of the structural hole, and a lower value for constraints. These scores indicated that the two clusters had strong agglomeration and diffusion functions in regional tourism flows. In culture-dominated clusters, C4—with its transportation advantage—developed as a core distribution center in the tourism flow network. Shanxi is long and narrow in the north and south, characterized by relatively long transportation distances.

**Table 3:** Hierarchical structure characteristics between clusters.

Category	Cluster	Percentage of in and out flows		Centrality degree			Structural hole		
		In	Out	Degree			In	Out	Degree
Nature-dominated	C 5	88.235	11.765	20	0	55.556	2.310	0.770	0.917
	C 9	67.692	32.308	30	0	58.824	2.608	0.652	0.841
Culture-dominated	C 1	50.000	50.000	40	0	62.5	2.616	0.523	0.861
	C 4	62.376	37.624	30	0	58.824	1.608	0.402	0.784
	C 6	66.667	33.333	80	9.444	83.333	7.428	0.825	0.605
	C 7	64.151	35.849	50	3.333	66.667	2.510	0.418	0.713
Nature-culture	C 2	77.011	22.989	100	31.667	100	7.353	0.668	0.555
	C 3	69.828	30.172	30	0	58.824	2.052	0.513	0.829
	C 8	58.929	41.071	50	0.556	66.667	4.120	0.687	0.710
	C 10	66.857	33.143	90	17.222	90.909	6.068	0.607	0.687
	C 11	60.606	39.394	20	0	55.556	2.479	0.826	0.807

### 4.3 Organizational patterns of tourism clusters

**Fig 7:**
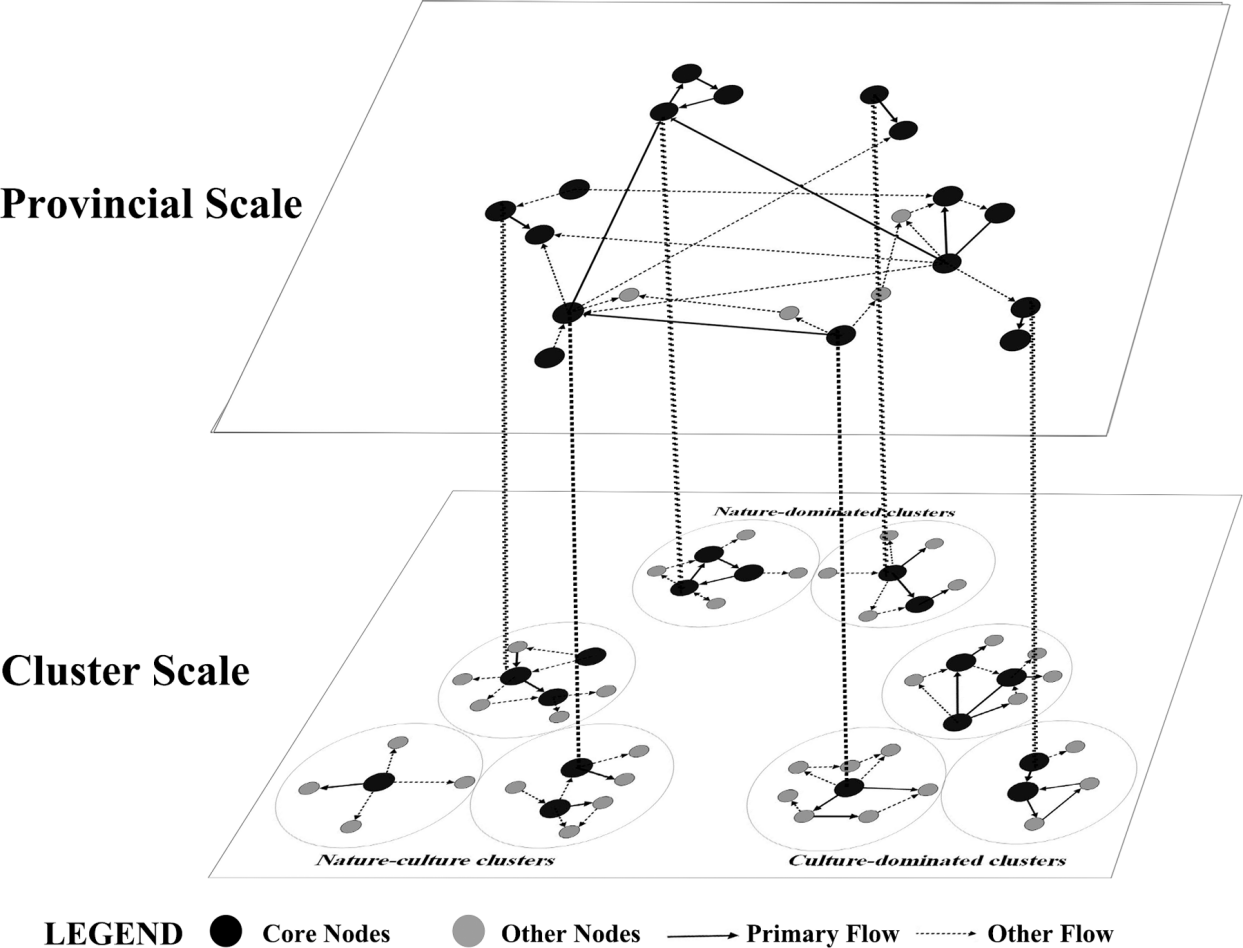
Organization pattern and hierarchical structure of tourism flow cascade network.

Based on our study’s results and a synthesis of previous findings, the organizational patterns within and between clusters in Shanxi Province were abstracted into a conceptual model (**Fig 7**). Based on the theory of tourism spatial structure, the number of core nodes, and links between nodes, these clusters can be classified into single-, dual-, and multi-core assemblage types. The *single-core* assemblage type considered the core destination as the main distribution center, and the other nodes relied heavily on its spillover effect. Dual-core assemblage clusters included the following two patterns: (1) Primary flow connected the two core nodes and formed a tourism growth pole. The other nodes relied on the development of the tourism growth poles. (2) Primary flow did not exist between the two core nodes. The primary flow within the clusters connected to one core node. The other core node was closely connected to the nodes in other clusters. Multi-core assemblage types had multiple growth poles and developed multiple axes of tourism development.

The organizational patterns among the three cluster categories exhibited major differences. Nature-dominated clusters developed only dual-core and multiple-core assemblage types. The most frequent travel route between nodes at close distances was a regional tour pattern. Tourists started at one node, visited multiple nodes sequentially, and then returned to that node. By contrast, among nodes at longer distances, the routes primarily exhibited the characteristics of a trip-chaining pattern. Tourists start at the core node and end at the exit node in the opposite direction. The node connections were predominantly unidirectional routes. Culture-dominated clusters not only had dual-core- and multi-core assemblage types but also a single-core combined type (C1). Additionally, culture-dominated clusters had developed fully circular touring (C4), whereby a wide range of multiple destinations were visited. Tourists began from Liuxiang or Jinci and followed a non-repeating circular route to visit all the nodes within the cluster. This excursion route was characterized by a larger scale of radiation from the core nodes to the edge nodes via transit nodes, with broader and stronger connections.

At the level of inter-cluster connection, a large difference prevailed in the control ability over the provincial tourism flow network of the three categorization clusters. Multi-core assemblage in culture-dominated and nature-culture clusters were primarily located in cities and had the advantages of convenient transportation and optimal tourism service facilities; thus, they predominantly developed into the region’s core, with greater radiating capacity and strong control over the tourism flow network and other associations. Although the nature-dominated clusters had distinctive and high-quality tourism resources, they were limited by their reception capacity and accessibility. Thus, these clusters developed radial nodes in the tourism flow network.

## 5 Discussion

### 5.1 Mechanisms of formation of heterogeneity in cluster networks

The Heterogeneity in the network of different motivated tourist flows is related to the distance between tourist destinations. The law of distance decay suggests that a tourist’s demand for a destination is inversely proportional to the increase in time, money, or effort required to reach a location [38]. Shanxi is long and narrow in the north-south direction, with relatively long transportation distances. The distance between nature-type scenic spots is relatively far, thus the “polarization” effect of the core nodes is more significant. Culture-type and culture-nature-type tourism flow networks, the distance between nodes is relatively close, thus, the frequency of association between its nodes is higher and the radiation effect is more obvious.

The heterogeneity in the network of different motivated tourist flows is related to the origin of tourists. Short-haul tourists have relatively homogeneous motivations and tend to choose a single type of destination, such as the movement patterns of short-haul nature-based and short-haul exploratory historical and cultural tourists [18]; long-haul tourists experience a large degree of climatic and cultural differences between their places of origin and their destinations, and therefore seek to take part in multi-destination trips to prolong their length of stay [1,19,39], such as the travel patterns of long-haul multi-interest travelers [18] and non-resident travel patterns [33].

The hierarchical heterogeneity in the network of different motivated tourist flows may be attributable to the differences in socioeconomic status and amenities between clusters. The development of transportation, infrastructure, and service facilities at tourism destinations profoundly affects tourists’ travel patterns [25,35]. Regions with favorable locations and well-developed infrastructure and services are more likely to attract tourists because they reduce their perceptions of the risks involved in the destination [16,40]. Nature-culture clusters primarily have a long development history, such as the Datong (C1) and Taiyuan (C7) clusters, which have superior transportation locations and optimal facilities. These clusters primarily serve as the first stop for tourists to enter Shanxi and develop into the tourism flow network’s core distribution centers, which have a strong function of gathering and spreading the regional tourism flow. The nature-based clusters are relatively inaccessible and have relatively poor facilities; thus, their connections are highly concentrated within their clusters, and fewer tourists use them to transit or travel to other clusters.

The hierarchical heterogeneity in the network of different motivated tourist flows is also associated with destination promotion. Tourists are often more inclined to conduct excursions near the hotels they stay at, in contrast to more remote attractions that are less likely to be included in their excursion plans, unless the remote attractions themselves are part of the region’s iconic tourism brand [41]. This study further found that tourists shifting through different types of tourism flow networks would first shift among the core nodes of associations and use the new core of associations as a base for tours of neighboring scenic spots. These core nodes are mostly iconic tourism brands in the region, such as Pingyao Ancient City, Wutai Mountain, and Hanging Temple.

### 5.2 Managerial implications

In this study, considering the spatiotemporal behaviors of tourists as the main body of the flow-space movement, the differences in the hierarchical structure, geographical constraints, and agglomeration-diffusion effects among natural, cultural, and nature-culture tourist flow networks were compared in detail. This study’s results are of great importance for the design, planning, and management of sustainable and resilient tourism attraction clusters, which can ultimately improve the competitiveness of tourism destinations. This study’s specific managerial implications are as follows:

(1) For the continued development of tourism in Shanxi, breaking the constraints of the administrative system and regional division and actively promoting regional tourism cooperation are necessary. Tourists’ spatial movement in Shanxi is greatly restricted by topography, resource types, traffic conditions, and infrastructure. The boundaries of the clusters are not completely consistent with the boundaries of the administrative divisions. Each community—focusing primarily on internal connections—has relatively weak external connections. The local Government should firmly seize the policy opportunities for the coordinated development of national cross-border tourism areas—such as the “Yellow River Cultural Park,” “Great Wall Cultural Park,” and “Taihang Mountain Tourism Plan”—and create high-quality development-oriented boutique tourism belts. In particular, utilizing communities with strong network access capabilities (e.g., C11, C3, and C4) and simultaneously focusing on improving the access capabilities of some bridge-point spaces (e.g., C1) are critical.
(2) Further boosting the tourism consumption demands within Shanxi Province and focusing on improving the network access capabilities of edge nodes are vital. Shanxi’s tourist flow is highly concentrated in the core nodes and structural holes of the clusters. The development of edge and sub-edge nodes is challenging, particularly when improving the level of intelligence and facilities. They require governments to encourage policies and provide financial support. To guide the orderly flow of tourism, the government should build a “5G+” smart tourism management platform to accurately monitor risk prevention, regulation and guidance, emergency handling, and peak-period park-entry information. Furthermore, these nodes will be guided to upgrade facilities and equipment, products, and projects by gradually promoting the implementation of the strategy of upgrading sub–edge and edge nodes to higher-grade tourism scenic areas. Scenic spots should align with the rapidly changing consumption trends and launch and carry out a series of activities and consumption scenarios in various forms. These measures can improve the network accessibility of various associations.
(3) Through the flow advantage of the core nodes, Shanxi creates high-quality tourism routes cultural and natural nodes. Consistent with previous study’s results, the distance attenuation effect of composite clusters is significantly lower than that of cultural and natural clusters, the connection between the core nodes and other nodes is significantly closer, and the flow is richer, indicating that the multi-core network is still an effective initiative to maintain the flow [23]. The multi-core cluster generally has a more efficient cluster network and is conducive to alleviating problems such as traffic congestion owing to overtourism in the region [40,42]. Moreover, tourists prefer visiting routes with higher thematic diversity [36]. By launching a series of cultural and eco-tourism routes based on ancient architecture, colorful sculptures, and murals and featuring natural landscapes, the “core-edge-core” linked high-quality tourism routes will be constructed. This strategy brings traffic to edge tourism nodes without losing the attractiveness of the core nodes.

### 5.3 Theoretical implications and limitations

Based on geographic big data, this study utilizes association detection and complex network algorithms to explore the heterogeneity and organizational patterns of subgroup tourism flow networks, considering motivation as a segmentation criterion. This study found that (1) three types of tourism flow networks with different motives were identified, namely networks motivated by exploring nature, networks motivated by exploring history and culture, and networks driven by nature-humanity composite motives. (2) There are differences in agglomeration characteristics and geographic attenuation effects among the three types of clusters: natural clusters, cultural clusters, and natural-cultural clusters. (3) The tourism flow networks of the three types of motives present a multi-scale hierarchical nested pattern. This study analyzes the cross-scale spatial correlations of tourism flow networks from the unique perspective of motivation, enriching the understanding of the complex mechanisms of interaction between tourist behavior and the built environment.

To analyze the network structure of tourist flows with different motivations, from the guest position perspective is to explore the flow patterns of tourists between tourist attractions with thematic similarities. Previous studies have overemphasized the thematic differences between destinations, arguing that the combination of type-different scenic areas and services can effectively pull tourism development, and a large number of studies have been conducted [43,44]. And this study found that tourists will not only move between type-differentiated scenic spots, but also between thematically similar and geographically neighboring scenic spots. This phenomenon, which is in line with the geographic attenuation law that exists in the spatial movement behavior of tourists, also indicates that there is a compatible and synergistic relationship between thematically similar scenic spots. Therefore, another novelty of this study is the use of the network analysis paradigm to explore the spatial compatibility and synergy between thematically similar scenic spots and to deepen the understanding of the spatial structure of tourism sites.

This study has some limitations. First, although big data analytics provide opportunities to explore tourist mobility at the cluster scale, this study is limited by the use of online platforms that share data as a data source, which does not accurately reflect the behavior of all tourists. Online travel platform-sharing users are predominantly young people, and older and less-educated tourists may exhibit different behaviors at destinations [41]. In future research, we could reduce the impact of data representativeness and bias by combining big data and field survey data for interactive validation. Second, owing to data limitations, mobility patterns between subgroups of people with different attributes have not yet been compared. Future studies could aim to identify detailed patterns, for example, by exploring the heterogeneity of travel mobility patterns across population subgroups by age and gender. Finally, other factors—including resilience, stability, and other network structural characteristics—also influence tourists’ spatiotemporal behavioral patterns. Future work could further explore the spatiotemporal evolutionary process of the resilience of tourist flow networks. Despite these limitations, our study provides insights that differ from those of existing studies by considering the heterogeneity of the travel patterns of tourists with different motivations.
